# Deformation twinning evolution from a single crystal in a face-centered-cubic ternary alloy

**DOI:** 10.1038/srep11290

**Published:** 2015-06-10

**Authors:** Zhenyu Zhang, Song Yang, Dongming Guo, Boya Yuan, Xiaoguang Guo, Bi Zhang, Yanxia Huo

**Affiliations:** 1 Key Laboratory for Precision and Non-Traditional Machining Technology, Dalian University of Technology, Dalian 116024, China; 2 State Key Laboratory of Mechanical Transmissions, Chongqing University, Chongqing 400044, China; 3 Changzhou Institute of Dalian University of Technology; 4 Department of Mechanical Engineering, University of Connecticut, Storrs, CT 06269, USA

## Abstract

Deformation twinning evolution from a single crystal is conducted by molecular dynamics simulations, to elucidate a twinned face-centered-cubic alloy in an experiment with hardness up to 100 times as that of single crystals, and with ductility simultaneously. Critical twinning stress of cadmium zinc telluride (CdZnTe or CZT) calculated is 1.38 GPa. All the twin boundaries are along the (11-1) orientation, except the one with the (-111) plane that supports the indentation, interpreting the unidirectional and boundary-free characteristics, confirmed in the experiment. Three twin thicknesses after unloading are 3.2, 3.5, and 16 nm, which is consistent with the experimentally repeated pattern of a lamellar twin with thickness larger than 12.7 nm, followed by one or several twins with thicknesses smaller than 12.7 nm. An inverse triangle of a twin combining with three twins generate a synergistic strengthening effect through the hardening and softening functions, illuminating the ultrahigh hardness demonstrated in the experiment. Twinning takes place in loading, and detwinning occurs in unloading, which expounds the high ductility observed in the experiment.

Nanotwinned (nt) copper (Cu) exhibits unusual mechanical properties up to ten times as that of the coarse-grained counterparts, remaining the ductility, and therefore has received considerable attentions^1^,^2^. To further increase the mechanical properties of an nt metal, electrodeposition^1^,^2^, sputter deposition^3^, dynamic plastic deformation^4^, surface mechanical attrition treatment (SMAT)^5^ and surface mechanical grinding treatment (SMGT)^6^ have been developed. Nevertheless, during the past decade, no obvious improvements in the mechanical properties of nt metals are achieved. The strength of an nt metal still remains ten times of its conventional counterparts, as published in 2004^1^. Thereby, it is a challenge to develop a novel fabrication method to increase the mechanical properties of an nt metal, and to retain its ductility. On the other hand, most present nt studies mainly focus on the nt Cu^7^. However, the practical engineering materials are alloys, rather than pure metals. Hereby, the nt work of alloys is another challenge for their pragmatic applications. Nonetheless, an experiment on the nt face-centered-cubic (fcc) ternary alloy presently sheds light on solving the challenges^8^, in which the mechanical properties of the nt ternary alloy induced by nanoindentation is 100 times as that of a single crystal, maintaining the ductility^8^. Consequently, the nt ternary alloy reveals ultrahigh hardness and high ductility. However, the deformation twinning mechanism is not understood. This limits the development of fabrication methods for nt alloys. It is therefore extremely significant to explore the deformation twinning evolution from a single crystal in an fcc ternary alloy both scientifically and technologically.

It is difficult in an experiment to observe the evolution process of a metal from a single crystal to the nt structure at the nanoscale in a short time. Thus, molecular dynamics (MD) simulation is an effective approach to elucidate the deformation twinning mechanism in the fcc ternary alloy^8^. At present, most nt MD work is on the nt Cu^9^,^10^ and other single element metals^11^,^12^. The potential function of a single element metal could be usually found in the previous literatures, whereas that of an fcc ternary alloy is hard to be discovered in the previous reports, resulting in the scarceness of MD simulation for an fcc ternary alloy. The MD study on the ternary alloy with ultrahigh hardness and high ductility has not been reported^13^. Since the deformation twinning mechanism from a single crystal is not clear, an fcc binary single crystal is cracked under nanoindentation^13^, rather than forming twins from a single crystal. Because of the difficulty to form twins from a single crystal, investigations of deformation ordinarily begin from an established nt structure^9^,^10^, instead of a single crystal. Accordingly, it is a hard work to achieve the deformation twinning from a single crystal in an fcc ternary alloy.

How to fabricate nt alloys with ultrahigh hardness and high ductility from a single crystal directly impacts the pragmatic applications of nt metals, and it is therefore intriguing to explore the deformation twinning evolution from a single crystal in an fcc ternary alloy. In this study, deformation twinning evolution is achieved from a single crystal in an fcc ternary alloy using MD simulations, and critical twinning stress is obtained.

## Results

**Figure 1:**
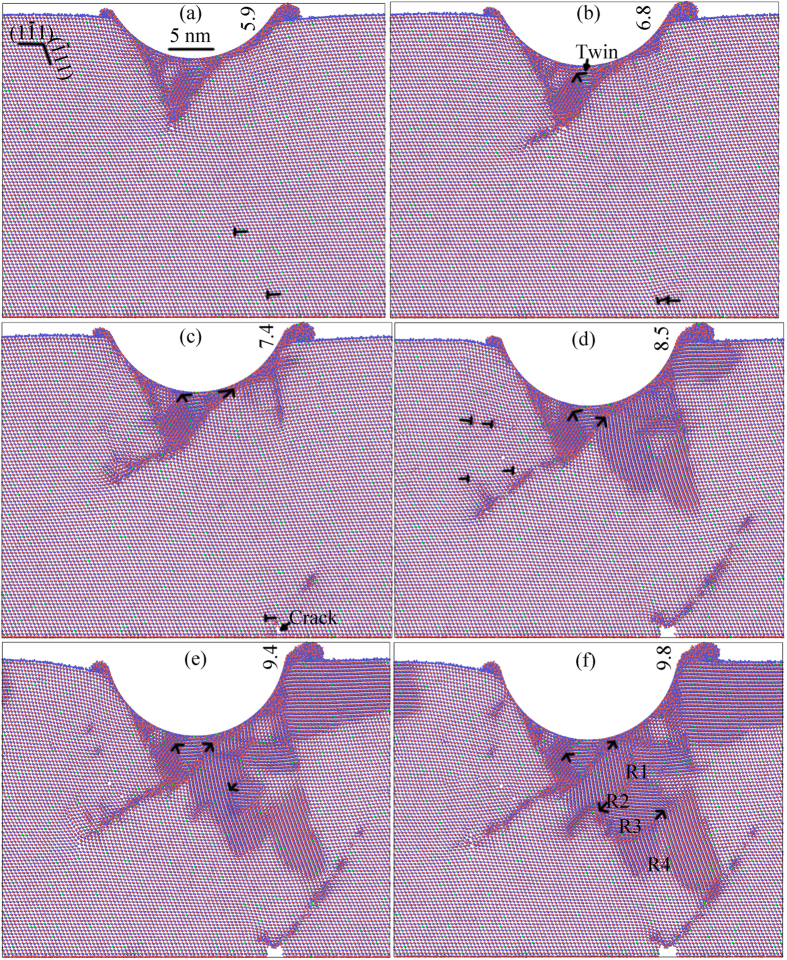
Snapshots of deformation twinning evolution at indentation depths of (**a**) 5.9 nm, (**b**) 6.8 nm, (**c**) 7.4 nm, (**d**) 8.5 nm, (**e**) 9.4 nm, and (**f**) 9.8 nm under the loading conditions used in the MD modeling from a single crystal for an fcc ternary alloy.

Figure 1 shows the snapshots of deformation twinning evolution at different indentation depths under the loading conditions used in the MD modeling from a single crystal for an fcc ternary alloy. The deformation is confined in an inverse triangular region induced from the indentation, as shown in Fig. 1(a), and pile-ups are formed on the surface of the MD model. Deformation is also transferred downward through both edge dislocations marked by turned “T” in Fig. 1(a) below the inverse triangle along the (-111) orientation. With an increase in indentation depth to 6.8 nm, the deformation is mainly in the inverse triangle, leading to the formation of a twin (Fig. 1(b)) within the inverse triangle with twin boundary along (-111) intersecting at the right side of the inverse triangle. At the same time, stress concentration is identified at the bottom in MD model confirmed by both overlapped edge dislocations along the (-111) orientation. A twin configuration is initially induced in Fig. 1(c), and a small crack emerges at the bottom along the (-111) direction. This is consistent with previous reports^13^, in terms of the impediment of slips along (-111) orientation, induced by the fixed atomic layer at the bottom in MD model. In Fig. 1(d), a twin along the right side of the inverse triangle with twin boundary in the (11-1) direction is produced. The crack found in Fig. 1(c) grows slightly bigger. Deformation is transferred to the left side by edge dislocations along the (1-11) orientation. Both deformed lines are observed on the (11-1) plane. At the indentation depth of 9.4 nm (Fig. 1(e)), a twin is induced below the inverse triangle with twin boundary along the (11-1) orientation. With the formation of the twin, the crack induced in Fig. 1(d) remains unchanged. In Fig. 1(f), R1, R2, R3, and R4 represent four various rectangles. R1 is at the highest position, close to the inverse triangle. R4 is at the lowest location, adjacent to the intersecting point between an extending line of the left side of the inverse triangle and the fixed atomic layer at the bottom in the MD model. Both R2 and R3 are in the middle between R1 and R4. A twin is produced with boundary along the (11-1) orientation between R3 and R4. Thus, there are four twins formed in Fig.1 (f), and their twin thicknesses are 3.8, 4.7, 5.9 and 10.4 nm, respectively along (-111) orientation. The increase in the indentation depth from 8.5 to 9.8 nm does not change the crack size and configuration, due to the formation of twins, leading to the release of stress. This is different from those published in previous literatures^13^, where cracks enlarge with an increase of indentation.

**Figure 2:**
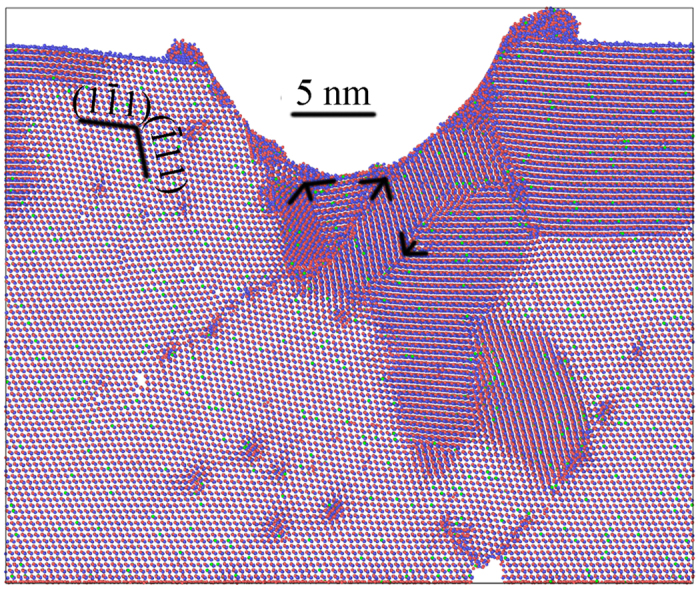
Snapshot of the MD model after unloading at an indentation depth of 10 nm from a single crystal for an fcc ternary alloy .

Figure 2 depicts the snapshot of the MD model after unloading at an indentation depth of 10 nm from a single crystal for an fcc ternary alloy. The twin generated between R3 and R4 disappears, indicating the occurrence of detwinning during the unloading process. The other three twins are left after unloading. The crack size and configuration observed in Fig. 1(f) keep unchanged, because of the detwinning giving rise to the release of stress.

## Discussion

**Figure 3:**
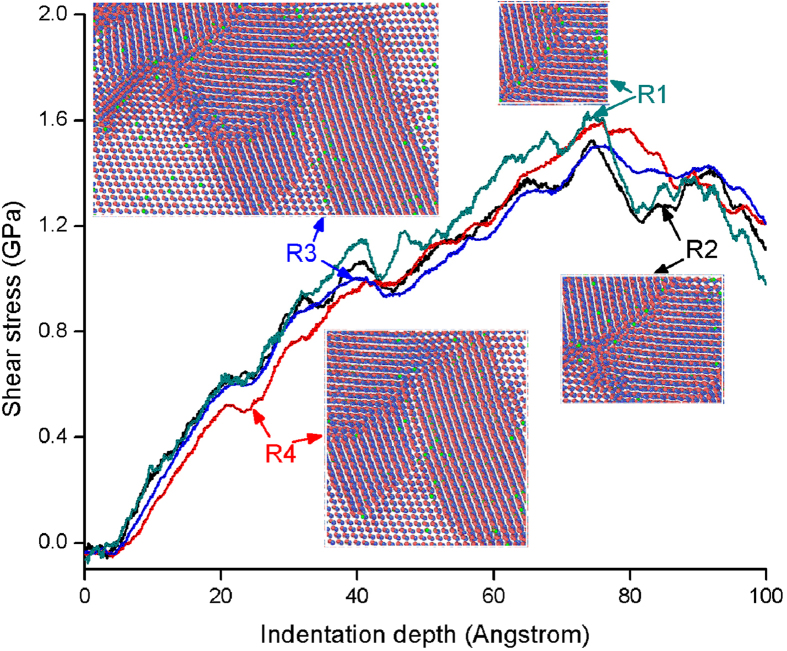
Shear stresses as a function of indentation depth for the four rectangles marked in Fig. 1(f).

The critical twinning stress, *τ*_*p*_ is expressed^14^:


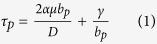


where μ is the shear modulus, γ the stacking fault energy, *b*_*p*_ the magnitude of the Burgers vector of the Shockley partial dislocation, α a constant related to the characteristics of dislocation, and *D* is the twin thickness between the adjacent twin boundaries. α is taken as 1.0^14^, and the average *D* is 6.2 nm according to the four twin thicknesses in Fig. 1(f). The stacking fault energy of CZT^8^ varies from 8 to 11.4 mJ m^−2^, and γ is taken as 10.0 mJ m^−2^. The magnitude of twinning partial Burgers vector, *b*_*p*_ is 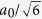
15. *a*_*0*_ is the crystalline lattice constant of CZT, taking 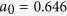 nm^16^ and 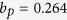 nm. *μ* is calculated^17^:


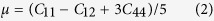


where *C*_*11*_, *C*_*12*_, and *C*_*44*_ are the elastic stiffness constants and given^18^:


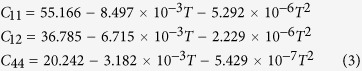


where the unit of *T* is °C. In this study, T is 26.85 °C, and *C*_*11*_, *C*_*12*_, and *C*_*44*_ are 54.938, 36.605, and 20.157 GPa, respectively. Therefore μ is 15.761 GPa, and the critical twinning stress of CZT, *τ*_*p*_ is 1.38 GPa. Figure 3 illustrates the shear stresses as a function of indentation depth for the four rectangles marked in Fig. 1(f). The shear stress calculated by MD at the six indentation depths corresponding to those in Fig. 1 for the four rectangles depicted in Fig. 3 is listed in Table 1. At the indentation depth of 5.9 nm (Fig. 1(a)), the shear stress in the four rectangles, as listed in Table 1, is less than *τ*_*p*_, revealing the absence of twin. At the loading speed of 100 m/s, the deformed area is confined within the inverse triangle in Fig. 1(a). With the increasing in the indentation depth to 6.8 nm (Fig. 1(b)), the shear stress in both R1 and R4 is more than *τ*_*p*_. The shear stress in R1 reflects that in the inverse triangle. A twin is therefore formed within the triangle. The shear stress in R4 represents that of the intersecting point at the bottom in MD model. Accordingly, there is stress concentration at the intersecting point. At an indentation depth of 7.4 nm, the shear stress in four rectangles reaches the maximum. The shear stress in R1 is the highest among four rectangles, which induces the formation of a twin (Fig. 1(c)) along the right side of the inverse triangle. The shear stress in R4 is higher than both R2 and R3, indicating the stress concentration at the intersecting point. This is attributed to the impediment of slip along the (-111) orientation, resulting in a crack shown in Fig. 1(c). For Fig. 1(d), the twin in Fig. 1(c) becomes obvious, and the crack grows slightly bigger. Slips also transfer leftward via edge dislocations, as found in Fig. 1(d). With the formation of crack and twin, the stress is released, leading to a reduction in the shear stress, as observed in Table 1 and Fig. 3. The shear stress in R3 is 1.4 GPa in Fig. 1(d), which is more than *τ*_*p*_, indicating the formation of a twin. When the indentation depth is increased to 9.4 nm, a twin is formed in R3, as shown in Fig. 1(e). The shear stress is reduced to 1.27 GPa with the formation of a twin in R3, and the crack does not show much change in its configuration and size as the indentation depth is increased from 8.5 to 9.4 nm. The shear stress in R3 is 1.38 GPa, as listed in Table 1, which is equal to *τ*_*p*_, predicting the formation of a twin in R3. It is confirmed in Fig. 1(f) with the indentation depth to 9.8 nm. With the decreasing shear stress in R4, the crack keeps unchanged basically with the formation of a twin in R3. Detwinning happens during the unloading process, as observed in Fig. 2.

All the twin boundaries formed in Fig. 1 are along the (11-1) orientation, except the one within the inverse triangle presented in Fig. 1(a). This elucidates the unidirectional and boundary-free characteristics, confirmed in the experiment^8^. Three twin thicknesses left in Fig. 2 with the twin boundaries along the (11-1) orientation are 3.2, 3.5 and 16 nm. This is in a good agreement with the experimental nt pattern, in which a twin with thickness larger than 12.7 nm, is followed by one or several twins with a thickness smaller than 12.7 nm^8^. The inverse triangle consisting of a twin is maintained after unloading, and the boundary of twin is along the (-111) direction, intersecting with another twin boundary with the (11-1) orientation. Both twin boundaries intersect within the inverse triangle, and the twin with the (-111) boundary terminates at the left side of the triangle. Furthermore, the lamellar twin with a thickness of 16 nm has a hardening effect, and the two twins of their respective thicknesses of 3.2 and 3.5 nm have a softening effect^2^,^8^,^13^. The inverse triangle consisting of a twin combining with three twins generate a synergistic strengthening effect with both hardening and softening functions, indicating the ultrahigh hardness exhibited in the experiment^8^. Twinning takes place in loading conditions, and detwinning occurs during unloading, which induces the high ductility verified in the experiment for an fcc ternary alloy^8^.

In summary, MD is used to simulate the deformation twinning evolution from a single crystal in an fcc ternary alloy. The critical twinning stress of the fcc ternary alloy is 1.38 GPa. The evolution is significant in understanding the ultrahigh hardness and high ductility demonstrated in the experiment for the nt fcc ternary alloy. It also sheds light on how to fabricate nt structure in an fcc ternary alloy with ultrahigh hardness and high ductility in nanoindentation. The study elucidates the deformation twinning mechanism from a single crystal in an fcc ternary alloy, which is beneficial to solving the challenges of the nt alloys for practical applications.

## Methods

A MD model was constructed for simulating the deformation twinning evolution for an fcc ternary alloy from a single crystal, cadmium zinc telluride (Cd_0.96_Zn_0.04_Te or CZT) of ultrahigh hardness and high ductility^8^. The model was with a size of 39.6 nm in length, 4.6 nm in width and 33.9 nm in height. The MD model was fixed at the bottom and had a thickness of 1.8 nm with 180,000 atoms. Periodic boundary conditions were applied to both the width and length directions of the model. Prior to nanoindentation, the MD model was relaxed at an isothermal-isobaric condition at 300 K for 100 picoseconds (ps). A time step of 1 femtosecond (fs) was used in all the calculations using the MD model. A cylindrical tip of 10 nm radius was used to indent the MD model at loading and unloading speeds of 100 m/s. Dwelling time between loading and unloading was 5 ps. A displacement-controlled mode was used in both loading and unloading conditions. The maximum indentation depth was 10 nm. In the MD model, red color represents Cd atoms, green indicates Zn atoms, and blue means Te atoms. The calculation of the MD model was according to the modified Stillinger-Weber (SW) potential, and total energy of *N* atoms in the MD model is expressed^19^:


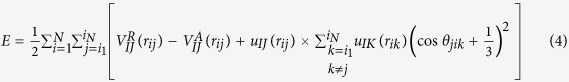


where *i*_*1*_,…, *i*_*N*_ is a list of neighbors of atom *i*, *θ*_*jik*_ is the bond angle induced by atoms *j* and *k* at the site of atom *i*, *V*_*IJ*_^*R*^*(r*_*ij*_) and *V*_*IJ*_^*A*^*(r*_*ij*_) are, respectively, pairwise repulsive and attractive functions, *u*_*IJ*_*(r*_*ij*_) is another pair function, and subscripts *ij* and *IJ* indicate, respectively, the pair of atoms and the species of the pair of atoms. *V*_*IJ*_^*R*^*(r*_*ij*_), *V*_*IJ*_^*A*^*(r*_*ij*_), and *u*_*IJ*_*(r*_*ij*_) are determined^19^:


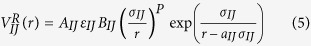



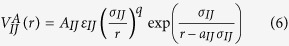



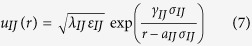


where *ε*, *σ*, *a*, *λ*, *γ*, *A* and *B* are seven pair-dependent parameters, and *p* and *q* are taken as values of 4 and 0, respectively. Note that *aσ* reveals the interaction range of the potential so that when *r* ≥ *aσ*, pair functions *V*_*IJ*_^*R*^*(r*_*ij*_), *V*_*IJ*_^*A*^*(r*_*ij*_), and *u*_*IJ*_*(r*_*ij*_) all vanish. Parameters of CZT SW potential are listed in Table 2^19^.

## Additional Information

**How to cite this article**: Zhang, Z. *et al.* Deformation twinning evolution from a single crystal in a face-centered-cubic ternary alloy. *Sci. Rep.* **5**, 11290; doi: 10.1038/srep11290 (2015).

## Figures and Tables

**Table 1:** Shear stress at six indentation depths illustrated in Fig. 1 for four rectangles depicted in Fig. 3.

**Indentation depth (nm)**	**5.9**	**6.8**	**7.4**	**8.5**	**9.4**	**9.8**
Rectangle R1 (GPa)	1.36	1.56	1.6	1.35	1.22	1.1
Rectangle R2 (GPa)	1.25	1.35	1.52	1.28	1.32	1.19
Rectangle R3 (GPa)	1.16	1.33	1.49	1.4	1.38	1.28
Rectangle R4 (GPa)	1.2	1.43	1.57	1.42	1.27	1.24

**Table 2:** Parameters of the Cd-Zn-Te SW potential (Energy in unit eV and length in unit Å)^19^.

**Pair** ***ij***	**ε**	**σ**	***a***	***A***	***B***
CdCd	1.182358	2.663951	1.527956	7.9170	0.767446
CdTe	1.385284	2.352141	1.810919	7.0496	0.886125
CdZn	0.690818	2.238699	1.812616	7.0496	1.010632
TeTe	1.849775	2.905254	1.594353	7.9170	0.730728
TeZn	1.546239	2.056363	1.907922	7.0496	1.255846
ZnZn	1.392961	2.367650	1.525521	7.9170	0.767628
